# Institutional capacity for health systems research in East and Central African Schools of Public Health: strengthening human and financial resources

**DOI:** 10.1186/1478-4505-12-23

**Published:** 2014-06-02

**Authors:** Daudi Simba, Aggrey Mukose, William Bazeyo

**Affiliations:** 1 Department of Community Health, Muhimbili University of Health and Allied Sciences, United Nations Road, P.O. Box 65015, Dar es Salaam, Tanzania; 2 Department Disease Control and Environmental Health, School of Public Health, College of Health Sciences, Makerere University, P.O. Box 7072, Kampala, Uganda; 3 Department of Community Health and Behavioral Sciences, School of Public Health, College of Health Sciences, Makerere University, P.O. Box 7072, Kampala, Uganda

**Keywords:** Capacity strengthening, Health systems research, Low-income countries

## Abstract

**Background:**

Despite its importance in providing evidence for health-related policy and decision-making, an insufficient amount of health systems research (HSR) is conducted in low-income countries (LICs). Schools of public health (SPHs) are key stakeholders in HSR. This paper, one in a series of four, examines human and financial resources capacities, policies and organizational support for HSR in seven Africa Hub SPHs in East and Central Africa.

**Methods:**

Capacity assessment done included document analysis to establish staff numbers, qualifications and publications; self-assessment using a tool developed to capture individual perceptions on the capacity for HSR and institutional dialogues. Key informant interviews (KIIs) were held with Deans from each SPH and Ministry of Health and non-governmental officials, focusing on perceptions on capacity of SPHs to engage in HSR, access to funding, and organizational support for HSR.

**Results:**

A total of 123 people participated in the self-assessment and 73 KIIs were conducted. Except for the National University of Rwanda and the University of Nairobi SPH, most respondents expressed confidence in the adequacy of staffing levels and HSR-related skills at their SPH. However, most of the researchers operate at individual level with low outputs. The average number of HSR-related publications was only <1 to 3 per staff member over a 6-year period with most of the publications in international journals. There is dependency on external funding for HSR, except for Rwanda, where there was little government funding. We also found that officials from the Ministries of Health often formulate policy based on data generated through *ad hoc* technical reviews and consultancies, despite their questionable quality.

**Conclusions:**

There exists adequate skilled staff for HSR in the SPHs. However, HSR conducted by individuals, fuelled by Ministries’ of Health tendency to engage individual researchers, undermines institutional capacity. This study underscores the need to form effective multidisciplinary teams to enhance research of immediate and local relevance. Capacity strengthening in the SPH needs to focus on knowledge translation and communication of findings to relevant audiences. Advocacy is needed to influence respective governments to allocate adequate funding for HSR to avoid donor dependency that distorts local research agenda.

## Background

The need for health systems research (HSR) to inform healthcare-related decision-making and policy making has long been underscored [1]. To enable HSR results to be used, however, their translation and communication is imperative [2]. Low-income countries (LICs) must begin to focus on how to improve evidence-based policy making by investing in HSR for better health service delivery and to achieve Millennium Development Goals [3,4]. The lack of an adequate understanding of how health systems are functioning in LICs compromises a country’s ability to propose solutions to existing problems [2]. The need for HSR was underscored in the Mexico Summit statement in a Ministerial meeting held in 2004 [5]. The need to strengthen capacity to conduct HSR to inform various stakeholders and organizations was echoed by the World Health Organization (WHO) in the first global symposium on HSR, held in Montreux, Switzerland, in 2010 [6].

Despite a clear appreciation of the role of HSR by policy makers in improving health systems performance, especially in LICs, academic and research institutions in these countries have limited capacity for HSR, a challenge that calls for deliberate interventions to build the required capacity [7]. Academic and research institutions are the desired recipients for research funding for HSR because they are perceived to have the capacity to prepare grant applications and implement them. Thus, strengthening the capacity of these institutions will enable them to better conduct HSR that is context-specific and can address local health systems challenges [8].

HSR focuses primarily on policies, organizations, and programs [9], with the ultimate objective of promoting coverage, quality, efficiency, and equity of health systems [1]. In terms of scope, HSR addresses any or several of the health system building blocks: human resources for health, policy and governance, financing, health information systems, service delivery, and medical technology and supplies [1]. Definitions of capacity often focus on the ability to perform organizational roles [10]. However, with HSR, there is a need to define capacity so that stakeholders have a common understanding when it comes to objectively identifying strategies necessary to address capacity problems [11]. While some schools of thought adopt a narrow definition of capacity for HSR that is limited to training, others broadly define it to include systems, processes, and networking at organizational and individual levels [6,11]. The capacity for HSR has also been defined as the level of expertise and resources needed for the production of new knowledge and its applications. This definition has been extended to include the capacity for research institutions to engage stakeholders in planning, managing, and financing activities to improve health [12].

Whereas the need for strengthening capacity for research in LICs has been emphasized in several WHO resolutions and global action agendas [9], implementation of a clear agenda and strategies for capacity development continues to be a challenge [2,6]. Progress is being made in both the production and providing funding to build capacity to undertake research for low- and middle-income countries, although locally this has grown at a much slower pace [9]. Hence, coordinated efforts are needed to strengthen capacity for HSR if LICs are to benefit from the funding available for HSR and assure achievement of their health systems objectives [6].

It is essential to have adequate data to inform HSR capacity development strategies and interventions for LICs; however, to date, there is only a limited amount available [6]. Some researchers have attributed weak capacity for HSR to inadequately skilled personnel and limited funding [6,12], and have called for more emphasis on improving human capacity and mobilizing additional financial resources for health research. An assessment of the resource gaps that exist in LICs is a critical starting point for any initiatives that may be proposed.

This paper reports findings from an assessment of the existing capacity to conduct HSR at seven schools of public health (SPHs) in six Central and East Africa countries, with a goal of identifying the priority areas that capacity development interventions must focus on. Specifically, this paper addresses whether these SPHs have adequate numbers of qualified staff to conduct HSR and disseminate its findings, and whether there exists organizational or institutional environments that support the same.

### Background

In 2008, seven SPHs in Central and East Africa came together to form the Higher Education Alliance for Leadership Through Health (HEALTH): Jimma University College of Public Health and Medical Science (CPHMS, Ethiopia); Kinshasa School of Public Health (KSPH, Democratic Republic of the Congo (DRC)); Makerere University College of Health Sciences, School of Public Health (MakSPH, Uganda); Moi University School of Public Health (MUSOPH, Kenya); Muhimbili School of Public Health and Social Sciences (MUSPHSS, Tanzania); National University of Rwanda School of Public Health (NURSPH, Rwanda); and University of Nairobi School of Public Health (SPHUoN, Kenya). It is out of the need to coordinate efforts towards building and strengthening HSR capacity across the region as well as promoting knowledge sharing across the institutions that the Alliance was formed. Realizing that they had similar objectives, the Future Health Systems (FHS) Research consortium [13] and the HEALTH Alliance came together in 2011 to form the Africa Hub. Africa Hub’s membership comprises the same SPHs that make up the HEALTH Alliance. The objectives of the Africa Hub are to (i) assess and strengthen capacity for HSR in the seven SPHs, (ii) extend networks for communicating learning in HSR and cross-country exchange of ideas and research, and (iii) improve capacity to communicate and promote uptake of research evidence in policy and decision-making. Since its inception, the Africa Hub has been supported by FHS.

In 2011, the seven SPHs undertook an assessment of the capacity for HSR that exists at the SPHs. The primary aim of the assessment was to identify where capacity development investments would have the greatest impact at each school. This paper, one in a series of four [14-16], presents findings on several topics: the available human resources and skill levels of staff to conduct HSR and disseminate its findings, and the environment within each school that supports HSR.

## Methods

### Design approaches

The assessment was conducted using self-assessments, key informant interviews (KIIs) of internal and external stakeholders, and a review of documents. Using three complementary approaches made it possible to enhance the validity of the data by subsequently triangulating data from the three sources. Multidisciplinary meetings of public health and management specialists, social scientists, statisticians, and epidemiologists were conducted to build consensus around key issues. These approaches were adopted because the primary intention of the assessment was to provide a systematic method for each SPH to reflect on its strengths and weaknesses with respect to HSR and to design effective strategies for strengthening HSR capacity. The detailed account on the design and methodological approach is reported elsewhere [16].

### Sampling strategy

Respondents for the self-assessment were purposively selected. They included staff that were teaching in the SPHs and had reported an interest in HSR and published or taught health systems-related courses (Table 1). Similarly, key informants from within and outside the SPH from key government institutions and agencies were purposively selected.

**Table 1:** Numbers of respondents interviewed in the study

**School of public health**	**Faculty/staff involved in self-assessment**	**Number of external stakeholders interviewed**
CPHMS, Ethiopia	26	6
KSPH, DRC	35	26
MakSPH, Uganda	15	6
MUSOPH, Kenya	22	15
MUSPHSS, Tanzania	16	4
SPHUoN, Kenya	5	12
NURSPH, Rwanda	4	4
**Total**	**123**	**73**

CPHMS, Jimma University College of Public Health and Medical Science, Ethiopia; KSPH, Kinshasa School of Public Health, Democratic Republic of the Congo; MakSPH, Makerere University College of Health Sciences, School of Public Health, Uganda; MUSOPH, Moi University School of Public Health, Kenya; MUSPHSS, Muhimbili School of Public Health and Social Sciences, Tanzania; NURSPH, National University of Rwanda School of Public Health, Rwanda; SPHUoN, University of Nairobi School of Public Health, Kenya.

### Data collection procedures

#### Self-assessment tool

A self-assessment tool was prepared from an instrument that the International Development Research Centre of Canada uses to assess the organizational capacity needs of its partner research organizations and another tool developed by the Canadian Health Service Research Foundation that seeks to examine the capacity of organizations to acquire and apply research. After developing the self-assessment tool, it was adapted by the team in a plenary meeting. The questions were categorized according to health system building blocks [1], and responses were scored using a 5-point Likert scale (ranging from 1 = strongly disagree to 5 = strongly agree). The tool was designed to assess organizational capacity for HSR rather than individual researchers’ capacity. Self-assessment questions focused on respondent’s opinions on the adequacy of academic members of the SPH to engage in HSR. Other questions asked about the availability and access to research funding for HSR, the external and internal organizational environment under which research is conducted, and individual researchers’ motivation to conduct research. Each SPH compiled the responses and presented them in a plenary meeting held as a forum to validate the findings. A detailed account of the content, administration, and limitations of the self-assessment tool can be found in articles published elsewhere [17-20].

#### Key informant interviews

KIIs were held with the Deans of the SPHs, academic members of staff within the universities, and key stakeholders from government ministries, bilateral and multilateral organizations, and non-governmental organizations (Table 1). Although, interviews were not transcribed, the interviewers took notes that were later analyzed. An interview guide was used with questions focusing on contextual factors influencing the conduct of HSR within the country and specifically at the respective SPH, existing policies, available human and financial resources, and staff motivation for HSR.

#### Document review

A review of relevant SPH and University documents was conducted to obtain general information about the organization, staff numbers and their qualifications, number of HSR publications, and research financing. A checklist was designed to collect relevant data on the number of staff working on HSR, their skills, availability of funds for HSR, and the type of research work being done.

### Data analysis

Quantitative data was captured electronically using Microsoft Excel software. The responses on the questionnaire were scored using a 5-point response scale with “strongly disagree” scoring a 1 and “strongly agree” scoring a 5. An average score was calculated for each response for each school using the formula:

$$\begin{array}{ll} \mathrm{Average}\;\mathrm{score}= & \left(\left[a\times 1\right]+\left[b\times 2\right]+\left[c\times 3\right]+\left[d\times 4\right]\right)\\ & \left(+\left[e\times 5\right]\right)/\left[a+b+c+d+e\right] \end{array}$$

Whereby:

a = number of respondents who strongly disagreed

b = number of respondents who disagreed

c = number of respondents who neither agreed nor disagreed

d = number of respondents who agreed

e = number of respondents who strongly agreed.

Content analysis was used to manually analyze qualitative responses, and categorize them into emerging themes and subthemes. The themes evolved around the existence of human resource policy, staff motivation for HSR at individual and institutional levels, and the existence of both policy for financing HSR and sources of funds.

### Ethical considerations

Ethical approval to conduct this study was sought and granted by the institutional Ethics and Research Committee of each university, except at MUSPHSS where the assessment was regarded as part of an ongoing routine capacity strengthening effort. Written informed consent was voluntarily obtained from respondents. In order to ensure confidentiality and anonymity, names of respondents were omitted from the study tools as well as in the analysis and dissemination of the findings.

## Results

A total of 123 academic staff members from the seven HEALTH Alliance’s SPHs participated in the self-assessment and 73 key informant interviews were conducted (Table 1). KSPH, DRC, contributed the largest number of participants (35,28.5%) and NURSPH, Rwanda, contributed the least (4,3.3%).

### Capacity of faculty to conduct HSR

#### Academic staff numbers

The total number of academic staff in each SPH varied widely, from 18 in NURSPH, Rwanda, to 113 in CPHMS, Ethiopia. The proportion of academic staff with PhDs in the SPHs ranged between 4.4% (5/113) in CPHMS, Ethiopia, to 67.0% (29/43) in MUSPHSS, Tanzania. At every SPH, a majority of the academic staff were male. For example, MUSPHSS, Tanzania, and MakSPH, Uganda, had only 26.7% (12/43) and 37.9% (22/56) female staff, respectively. While the majority of academic staff were over 45 years of age at MUSPHSS, Tanzania (63%; 27/43), only 20% (3/15) comprised this age group at MakSPH, Uganda.

#### Academic staff skills

Except for SPHUoN, Kenya, and NURSPH, Rwanda, respondents in the SPHs felt strongly that their school has individuals who can provide leadership for and have an interest in HSR (Table 2). Respondents in most SPHs agreed that their SPH has adequate numbers of academic staff with strong quantitative and qualitative skills that are important for HSR. Respondents in mostof the SPHs agreed that staff in their SPH possess adequate knowledge to teach HSR. Respondents in most SPHs felt strongly that their SPH has the ability to produce high quality proposals that could be funded and faculty that have the skills to write publishable papers on HSR topics. However, the perceived ability to write these papers did not match with the number of HSR publications in peer-reviewed journals (compare Tables 2 and 3). In most SPHs, HSR is conducted at the individual rather than the institutional level. Commenting on the reasons for the low HSR outputs in the SPHs, a respondent from one research institute reported:

**Table 2:** Respondents’ perception of staff skills for conducting health systems researchin their respective schools

**Opinion**	**Average score in the respective school of public health**						
	**CPHMS**	**MakSPH**	**KSPH**	**MUSPHSS**	**SPHUoN**	**MUSOPH**	**NURSPH**
I feel confident that there are individuals in this college who can provide high level leadership for HSR.	4.04	4.67	4.2	4.25	2.33	4.38	3.0
There are an adequate number of researchers in this college who are interested in HSR.	4.19	4.00	3.9	3.69	2	3.89	3.0
This college has adequate number of individuals with strong quantitative research skills who are interested in applying them to HSR.	3.81	4.2	3.8	3.8	2	3.8	4.5
This college has adequate number of individuals with strong qualitative research skills who are interested in applying them to HSR.	3.31	3.9	3.2	3.6	2	3.4	2.5
Staff in this college have adequate knowledge to teach HSR.	3.54	4.07	4.0	3.69	4	3.63	3.0
This college is able to produce high quality proposals that lead to funded grants for HSR.	3.58	4.67	4.2	4.19	2.33	4.0	2.5
Researchers at this college have the skills to write publishable papers based upon HSR that they conduct.	3.81	4.13	4.1	4.13	4	3.88	1.0
This college has a strong communications staff and capacity to effectively communicate HSR findings to manydifferent audiences.	3.19	3.40	3.9	3.13	1.67	2.62	2.0

**Table 3:** List of health systems research products (2005–2011)

**Research outputs**	Schools of public health^*^					
	**CPHMS**	**MakSPH**	**KSPH**	**MUSPHSS**	**MUSOPH**	**NURSPH**
Peer-reviewed articles in international journals	78	58	0	50	2	6
Peer-reviewed articles in national or local journals	103	0	1	16	2	28
Briefing notes (policy briefs)	27	7	0	0	1	0
Reports to agencies (Ministry of Health, donors learning materials, etc.)	6	3	12	20	1	4
Press releases and media briefings (includes radio and print)	6	1	0	0	1	0
Multimedia products (e.g., videos, blogs, radio spots)	2	0	0	0	0	0
Number of staff	113	56	41	43	20	9
Average number of peer-reviewed articles per person	1.6	1	<1	1.5	<1	3.8
Average number of total publications per person	1.2	1.2	<1	2	<1	4.2

* *Data from SPHUoN not available.*

*“People are not open to allow their institutions to do research, they are protecting something … they do not understand the value of research”* (KII, Research Institution, Kenya)

Respondents in most of the SPHs either were unsure or felt that their school lacked staff with capacity to effectively communicate HSR findings to different audiences such as media and policymakers (Table 2). Only KSPH, DRC, respondents strongly felt that their school has the capacity to communicate HSR results effectively to outside audiences.

#### Staff publications

The average rate of publications in peer-reviewed journals in the SPHs was quite low. The average publication rate for the past 6 years ranged from less than one per staff in KSPH, DRC, and MUSOPH, Kenya, to an average of approximately four per staff member in NURSPH, Rwanda (Table 3). At MUSPHSS, Tanzania, for example, despite having 29 staff members with PhDs, the average number of publications in peer-reviewed journals was 1.5 per person. Even after combining technical and consultancy reports as outputs, the ratio in most of the schools did not go beyond one output per staff. Over half of the publications were made through local journals at CPHMS, Ethiopia, and NURSPH, Rwanda, accounting for 57% (103/181) and 82% (28/34), respectively. At the MUSPHSS, Tanzania, and MakSPH, Uganda, academic staff preferred publishing in international peer-reviewed journals more than in local journals.

KIIs found that HSR stakeholders from outside the universities felt that research conducted by SPHs does not address issues of immediate need and relevance to policymakers and decision-makers. In addition, key informants noted that researchers do not communicate HSR results in a manner that is easy for policymakers to understand and use. Indeed, one respondent asserted:

*“Good research is going on in Kenya but it is gathering dust in shelves in the form of publications and theses … Publications are just used for career development by University lecturers thus indicating the lack of translation of research to care* (KII, MoH, Kenya)

### Contextual factors influencing the ability to conduct of HSR

#### Existence of human resource policy

All of the SPHs have human resource policies that spell out the teaching, research, and consultancy responsibilities of academic staff. Interviews with respondents revealed that although human resource policy documents are available both electronically and as hard copies located in the library and within the departments, staff members do not access the documents. Administrations take little initiative to ensure that all staff have read these policies. Most respondents reported ignorance on the existence of such policies, as reported by one of the respondents:

*“I think the biggest weakness in this university is that most policies are not known to staff. Myself, I am privileged because I have been in leadership therefore I have had opportunity to hear about what the policies are or get access to some of these policies because a need has arisen somewhere but otherwise most staff are not aware of some of the standing policies in the university. Some of the policy documents are posted on the intranet, but it isonly when one wants to find something about a specific policy that is when they go to the intranet to search”* (KII, MakSPH, Uganda).

With the exception of MUSOPH, Kenya, the SPHs reported having no formal research agenda that spells out priority areas for HSR. More often, research agendas are based on the priorities of the funding agency and less on the priorities of the SPH. Most of the seven SPHs reported having staff development and promotion policies, although in some schools the policies are controlled from outside the University. For example, Muhimbili University has a staff development and promotion policy, but staff remuneration is controlled by the Public Service Department, which is under the President’s Office. In all of the SPHs, project-related remunerations are guided by mutual agreements made between the recipient school and the respective donor.

All of the SPHs recommend recruiting people with PhDs. Some of the SPHs also allow recruitment of staff with Masters degrees in Medicine, because it is regarded as equivalent to a PhD. Muhimbili University, for instance, will consider applicants for faculty positions who have a Masters degree in Medicine from a 3- or 4-year training program. Applicants holding a Masters in Public Health from a 1- or 2-year program are rarely considered for faculty positions. This policy decreases the available pool of potential applicants because, except for MakSPH, Uganda, and MUSPHSS, Tanzania, the SPHs do not offer Masters degree courses lasting for more than 2 years. In elaborating this fact, a respondent in MUSPHSS, Tanzania, reported that:

“*These schools have to rely on PhD graduates who are not readily available in the market. However, this is not easy because most PhD holders in public health are attracted to NGOs and international organizations that pay relatively better salaries and remunerations*.” (KII, MUSPHSS, Tanzania)

#### Staff motivation to conduct research

Qualified members of staff are usually able to attract research grants and consultancy opportunities. Projects pay staff members according to the proportion of time contributed to a particular project. Job security is ensured by the nature of public employment, where rarely do staff get terminated or sanctioned for being unproductive. In addition, there are opportunities for staff to engage in research and consultancies that assures them an additional income. Low staff turnover rates provide additional evidence of good job security. At MUSOPH, Kenya, only three academic staff left in the past 10 years, while at MakSPH, Uganda, a turnover rate of 0.1% per year was reported. Only CPHMS, Ethiopia, reported a higher turnover rate where, in 1 year, 10 (3%) academic staff left the school; this turnover rate might seem to be low, but losing senior and experienced staff is a significant loss withwider implications. The main reasons for leaving included better salaries offered by NGOs, overseas and private institutions, and other universities that have better locations. The factors that influenced the retention of staff included regular and consistent promotion compared to other public servants, opportunities for further studies, the honor associated with working at university, and career development offered through participating in research projects.

#### Institutional support for HSR

All respondents agreed that the SPHs place a high priority on research, although some of the respondents highlighted that their schools do not emphasize carrying out original research, especially HSR (Table 4). With the exception of SPHUoN, Kenya, MUSOPH, Kenya, and NURSPH, Rwanda, faculty in other SPHs were confident that their schools provide adequate technical and scientific support for staff to develop and write research proposals. However, a majority expressed doubt on the adequacy of administrative support including budgeting and financial management for staff to develop and write research proposals (Table 5).

**Table 4:** Organizational support to motivate academic staff to engage in health systems research

	**Mean scores from institutions**						
	**CPHMS**	**MakSPH**	**KSPH**	**MUSPHSS**	**SPHUoN**	**MUSOPH**	**NURSPH**
The school of public health places a high priority on the conduct of original research.	3.92	4.53	4.1	3.88	2.33	4.50	4.5
Our school of public health places a high priority on health systems research.	3.35	4.27	3.6	3.40	4	4.13	2.5

**Table 5:** Perception of academic staff about the organizational support to motivate staff to engage in HSR

	**Mean scores from institutions**						
	**CPHMS**	**MakSPH**	**KSPH**	**MUSPHSS**	**SPHUoN**	**MUSOPH**	**NURSPH**
Development and writing of research proposals (in terms of technical and scientific support).	4.00	4.00	4.3	4.13	1	3.5	2.0
Development and writing of research proposals (in terms of administrative support).	3.46	3.13	3.8	3.69	1.33	2.5	3.0
Budgeting and financial management.	3.27	3.60	3.9	3.63	1.33	2.89	3.0
Publication of research in peer reviewed journals.	3.15	3.87	3.8	4.13	1	3.5	2.5

#### Policies on financing HSR

None of the SPHs has a policy for financial resource mobilization. As public institutions, each SPH draws recurrent and development funds from the government, thus, policies influencing appropriation and utilization of funds are part of public financing policies developed by the Ministries of Finance. The Universities are empowered to mobilize resources from external sources through research, consultancies, student tuition fees, and short courses. The fee structures, however, are controlled by the central government whose support to University is often limited. This leaves little room for the SPHs (or universities) to mobilize additional funds by increasing school fees. The only other flexible source of funding is through donor-funded research and consultancies. Respondents from five SPHs (except for NURSPH, Rwanda, and SPHUoN, Kenya) reported that their schools have the ability to produce high quality proposals that can win funding. They reported, however, that inadequate entrepreneurship skills among staff members limit their ability to mobilize resources from sources other than research grants.

#### Source of funds for financing HSR

Respondents from all the SPHs strongly disagreed that their government provided flexible funding. Although governments in the respective countries have pledged to provide about 1–2% of GDP for research activities, a majority of respondents from SPHs were unsure of the amount and the beneficiaries. Therefore, most SPHs depend on donor agencies for research funding. This consequently influences the type of research that is conducted (more often than not, research is donor driven). Reporting on availability of funds that could be used for HSR, a respondent at one SPH reported:

“*In spite of placing high priority on conducting original research, KSPH, DRC, has not focused its commitment on health systems research. The situation can be explained by the scarcity of funding opportunities and the fact that the HSR field is new*”. (KII, KSPH, DRC)

Data on the proportion of the SPH budget allocated for HSR were unavailable in most of the SPHs. The only school that was able to provide such data was CPHMS, Ethiopia, where it was reported that only about 1–2% of the University budget was allocated to HSR. At MUSPHSS, Tanzania, analysis of budget allocations for research could not be done because financial data are aggregated at the University level. However, from the university aggregated data, grants for research from donors constituted about half of the total University budget allocation (50.5%). For Makerere University, Uganda, a very small amount of funds are allocated to its SPH for purposes of capacity strengthening. The assessment also found that funding for HSR varied from $250,000 (US) per year in NURSPH, Rwanda, to $16 million (US) per year at MakSPH, Uganda.

## Discussion

This study has shown that, at most SPHs, adequate numbers of researchers exist with the quantitative and qualitative skills necessary for conducting HSR and writing publishable papers on their results. The findings are not surprising; it has been reported that, in Africa, people with PhDs in health and related fields account for 26% of the total workforce compared to that in Asia (20%) and in America (14%). This suggests that training in health and related fields is not the main challenge in Africa relative to other regions of the world [10]. The existence of an adequate number of highly qualified researchers might be because public employment in SPHs within these countries provides job security and various opportunities for faculty to engage in consultancies and research activities, which provide additional personal income. Job security might, however, also act as a disincentive for staff to strive for excellence.

Despite the capacity for conducting HSR in SPHs, the average number of publications per faculty member was low. It should be noted that, oftentimes, academic staff are involved in consultancy work or commissioned studies that may not always be reported to the SPHs. Additionally, most consultancy reports are rarely published because of ownership and ethical issues. We therefore acknowledge that findings on staff outputs may be an underestimation of actual staff outputs. However, we can draw a firm conclusion that the publication ratio in peer-reviewed journals was quite low. This can partly be attributed to an inadequate or complete lack of local funding, which reduces the opportunity for staff to conduct research. It could also be attributed to limited demand for research from Ministries of Health, because they appear to rely more heavily on information produced from *ad hoc* technical reviews and consultancies [6]. In addition to providing prompt information, these *ad hoc* reviews tend to be relevant to the issues at hand. Unfortunately, the quality of the data is often dubious, since quality is often compromised in the quest for urgent information at minimal cost. Many technical reviews often rely on grey literature and a rapid assessment in only a few purposively selected districts or facilities.

Due to relatively low pay in public universities, researchers are attracted to consultancies that consequently divert their attention to short-term projects and consultancies conducted at the individual level rather than longer-term collaborative research contracts [21]. Dependency on short-term arrangements undermines the development of longer-term relationships between researchers and policymakers, which is likely to undermine research uptake [22].

Over half of the publications at MUSPHSS, Tanzania, and MakSPH, Uganda, were through international peer-reviewed journals and rarely published in local journals. This could partly be explained by the fact that most research is donor driven and led by external researchers, who often become the first authors. It is thus not surprising that even the research agenda in these publications reflect areas of worldwide interest rather than themes that address issues of local interest. This finding concurs with studies conducted elsewhere in which the majority of HSR studies in low-income countries (LICs) had lead authors from high-income countries and only 4% lead authorship from the participating LICs [9].

We also ascertained that governments of the Africa Hub member institutions do not provide sufficient research funds for HSR to the respective SPHs. Most of the research projects were funded by international donors, according to key informant interviews and the self-assessment questionnaires. Our study showed a paucity of policy and media briefs generated from most SPHs. This is not surprising because research outputs from donor-driven research are more likely to be channeled through international journals, especially when the first authors come from developed countries. Since the findings in these publications do not address issues of immediate local relevance, local researchers have little or no incentive to prepare policy and media briefs for local consumption. Limited funding for research from governments has also been reported in other low-and middle-income countries [23], with only Brazil and Cuba reported to allocate about 2% of health expenditures to health research. Consequently, the interests of donors prevail, which increases the likelihood that the health research agenda and capacity-strengthening priorities in LICs will be distorted [23]. Failure of governments to fund HSR could be attributed to low budget allocations to the relevant ministries because funding is limited. For example, many African countries have failed to meet the Abuja Declaration in which member countries made a commitment to allocate 15% of their budget to the health sector by 2015 [24]. Thus, the lack of funding for HSR may be the result of inadequate financial capacity of the governments to sustainably fund health research projects, rather than because the government views health research as a low priority. A study done in Pakistan reported that over 95% of the budget allocated to health research institutions is tied up in salaries and operating costs [25]. Inadequate financing of HSR is seen around the world, where the lack of funding for health research in LICs has been reported as “the 90/10 gap” meaning less than 10% of health research funds are spent on 90% of the world’s diseases [26]. It has been reported that although an enormous amount of funding has been devoted globally to HSR between 2008 and 2012 [9], less than 1% of health expenditure in LICs was devoted to HSR [7].

The opportunities for SPHs to utilize internal resources are limited. Although universities in the studied SPHs were reported to mobilize resources from external sources through short-term studies and consultancies, school fees and tuition are set by the central government. The rates are fixed and do not necessarily meet the needs of the respective school. Thus, the only flexible source of funding for HSR is donor funding. However, the inadequate entrepreneurship skills among staff members limit the ability of staff to mobilize resources from external sources. This hampers efforts by researchers to set an HSR agenda driven by local needs, which is an important prerequisite in implementing HSR.

Finally, this study found that researchers tended to work on HSR on an *ad hoc* and individualized basis, thus attracting only a limited number of grants, which are usually relatively small in size. However, it was also reported to us that merely increasing funding for HSR might not be a panacea to all of the HSR challenges if an inadequate capacity in human resources remains. A combination of inadequate financial and human capacity creates a vicious cycle [25]. Thus, in a situation where researchers in SPHs are working as individuals, even if more funding were to become available for HSR, it might not be used effectively. There is, therefore, a need for researchers to work in multidisciplinary teams to become more effective. As long as they continue to work in isolation, they will not build a critical mass or the synergy required to support strong research teams that could take advantage of continuously emerging opportunities for capacity strengthening [6].

## Conclusions

The fact that there exists adequate skilled staff for HSR in the SPHs in East and Central Africa is impressive. However, HSR conducted by individuals, fuelled by MoHSW’s tendency to engage researchers at individual rather than institutional level, undermines the capacity for researchers to produce adequate, timely, and relevant research findings to suit the demands for policy decision-making. This study therefore underscores the need for researchers in SPHs located in LICs to form effective multidisciplinary HSR teams, through which they could solicit adequate funding, from within and outside the respective countries, in order to conduct research of immediate and local relevance.

Capacity strengthening in the SPHs needs to focus on knowledge translation and communication of research findings to relevant audiences such as policy- and decision-makers, and the media. This may be achieved through training and mentoring to enable academic staff, especially those of junior rank, to participate in writing research grants, conducting research, and packaging and communicating the results to enhance research uptake.

Finally, advocacy is needed to influence governments in the respective SPH on the need to allocate funding for HSR in order to avoid donor dependency that distorts the local research agenda and thus ensure timely and relevant information to policy decision-makers.

## Abbreviations

CPHMS: College of public health and medical sciences, JimmaUniversity, Ethiopia; DRC: Democratic Republic of the Congo; HEALTH: Higher Education Alliance for Leadership Through Health; HSR: Health systems research; KII: Key informant interview; KSPH: Kinshasa School of Public Health, DRC; LICs: Low-income countries; MUSPHSS: University of Health and Allied Sciences, School of Public Health, Tanzania; MUSOPH: Moi University, School of Public Health, Kenya; MakSPH: Makerere University College of Health Sciences, School of Public Health Uganda; NURSPH: National University of Rwanda School of Public Health, Rwanda; SPHs: Schools of public health; SPHUoN: University of Nairobi School of Public Health, Kenya; WHO: World Health Organization.

## Competing interests

The authors declare that they have no competing interests.

## Authors’ contributions

DS and AM participated in the study design, data collection and analysis, interpretation of the data, and writing of the manuscript. WB participated in the analysis and interpretation of the data, writing, and review of the manuscript. All authors provided immense input, and read and approved the final manuscript.
